# Union of an intra-articular distal radius fracture after successive failures of three locking plates: a case report

**DOI:** 10.1007/s11751-012-0127-6

**Published:** 2012-01-26

**Authors:** S. K. Khan, C. Gozzard

**Affiliations:** 1 Department of Trauma and Orthopaedics, Derriford Hospital, Plymouth, PL6 8DH UK; 2 35 Fellsdyke Court, Sheriff Hill, Gateshead, NE10 9SB UK

**Keywords:** Intra-articular fracture, Osteosynthesis, Locking plate, Stress raisers

## Abstract

We report a case of a 30-year old male, who presented with a right distal radius intra-articular fracture complicated by compartment syndrome. He was treated with fasciotomies and fracture fixation with a 3.5 mm LCP (Synthes^™^), followed 7 days later by skin graft. Repeat radiographs 8 weeks later showed a break across the plate at the level of an unfilled screw hole over the fracture. He underwent exchange plating with a 2.4 mm LCP Distal Radius Plate (Synthes^™^). This revision was complicated by an infected wound dehiscence 2 weeks later requiring multiple procedures. Radiographs at 20 weeks showed broken distal screws. A second revision was performed. At 12 months, the fracture had healed clinically and radiologically, but the three distal screws had broken. We discuss the multifactorial failures of the these three attempts at osteosynthesis, and which factors helped achieve osseous union. We also discuss the literature on volar locking plate breakage and conclude with the recommendations to avoid this rare complication.

## Case report

**Fig. 1:**
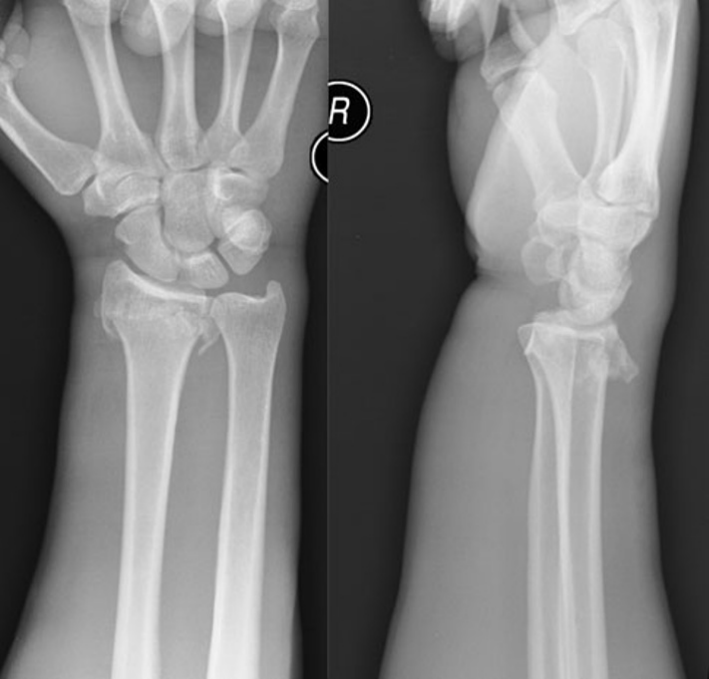
Radiographs after injury, showing the distal radius fracture

A 30-year old male presented to the Emergency Department after jumping over a small wall and falling onto his dominant right hand. He sustained a closed, dorsally angulated intra-articular fracture of his right distal radius (AO 23-C2) with extensive soft tissue swelling (Fig. 1). He was a skilled labourer with a past history of intravenous drug abuse, and ongoing mental health issues. He had smoked 20 cigarettes daily for the past 12 years.

The fracture was manipulated urgently under a haematoma block. One hour later, he started complaining of severe pain along the palmar aspect of the wrist. He had clinical signs of acute median nerve compression with worsening swelling. He was taken to theatre where he received emergent forearm fasciotomies along with a carpal tunnel decompression. The distal radius fracture was stabilised with a 3.5 mm locking compression plate (Synthes^™^, Solothurn, Switzerland). The distal Combi-hole was not filled as it was abutting the fracture site. The bone–plate contact was satisfactory, and augmentation with bone graft or bone substitute was not felt to be necessary. A ‘second-look’ procedure after 3 days revealed bruised muscle in the palmar compartment, but no obvious muscle necrosis. A split skin graft was applied during a third visit to theatre another 4 days later. The wrist was immobilised in a plaster splint after each of these procedures. He developed a superficial skin graft infection, which settled with 2 weeks of antibiotics.

**Fig. 2:**
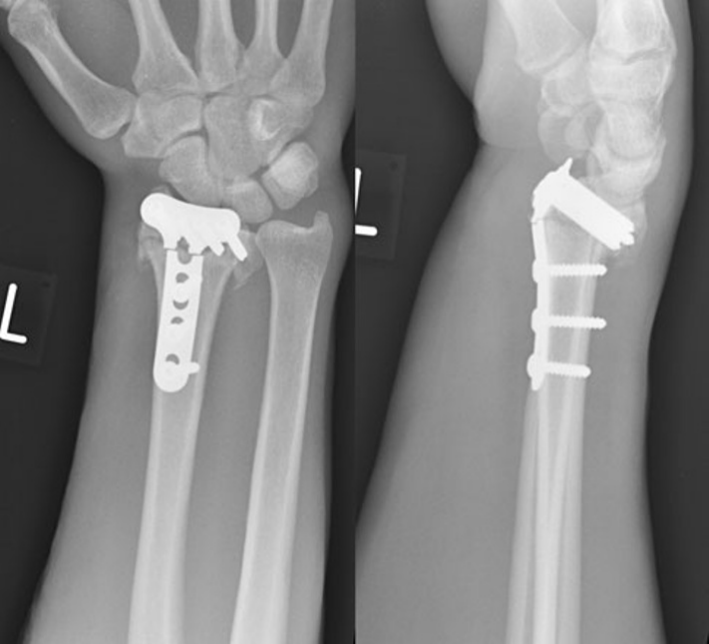
Radiographs at 6 weeks after the index procedure. Broken plate

When reviewed in fracture clinic 6 weeks post-operatively, he complained of worsening wrist pain. Radiographs revealed a transverse break across the plate, at the level of the distal Combi-hole, directly overlying the fracture site (Fig. 2). Routine blood investigations at this stage showed normal white cell count and C-reactive protein. There were no overt clinical signs of infection. The patient refused to have his fracture managed with an external fixator. He also refused to consent to iliac crest bone grafting at this stage. He received a revision fixation using a 2.4 mm LCP Distal Radius Plate (Synthes^™^, Solothurn, Switzerland), which has an 8-hole head configuration for the distal fragment. The plate was applied to the radial shaft with four bicortical screws, while four locking screws were inserted into the distal fragment. No macroscopic infection was seen in the bone or in the soft tissues intra-operatively. The wrist was immobilised in a plaster splint.

The patient was readmitted as an emergency 10 days later, with an infected wound dehiscence. This required two debridements and several negative pressure dressing changes using the VAC^®^ system (KCI^™^, San Antonio, TX, USA). He received intravenous antibiotics and a successful revision skin graft after a week. The wrist was immobilised in a full synthetic cast, and he was discharged home on oral antibiotics for a further 2 months.

**Fig. 3:**
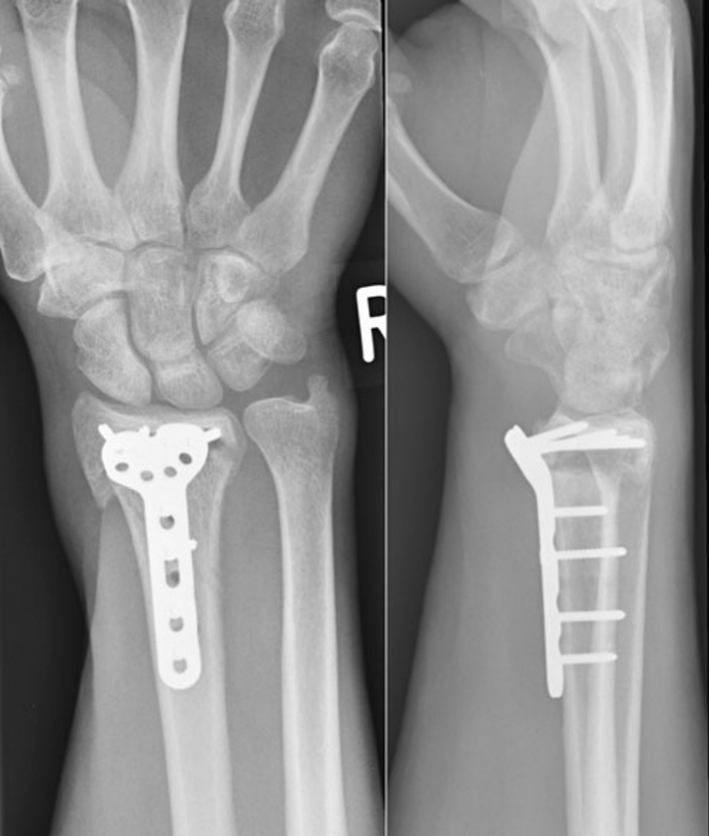
Radiographs at 20 weeks after the first revision procedure

**Fig. 4:**
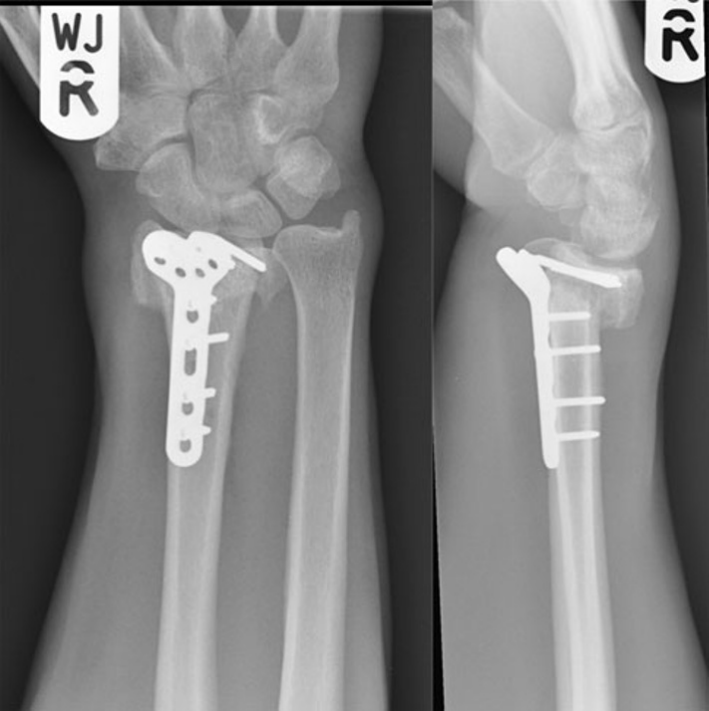
Radiographs at 26 weeks after the first revision procedure. Broken screws

He was followed up in clinic with serial radiographs, at 8, 14 and 20 weeks after the first revision osteosynthesis (Fig. 3). While these showed that the implant was still holding the fracture in an acceptable position, there was no discernible callus. At 26 weeks, his wrist was still painful and looked deformed. Radiographs at this appointment showed that the revision fixation had failed, this time with breaks across all the distal fragment screws (Fig. 4).

**Fig. 5:**
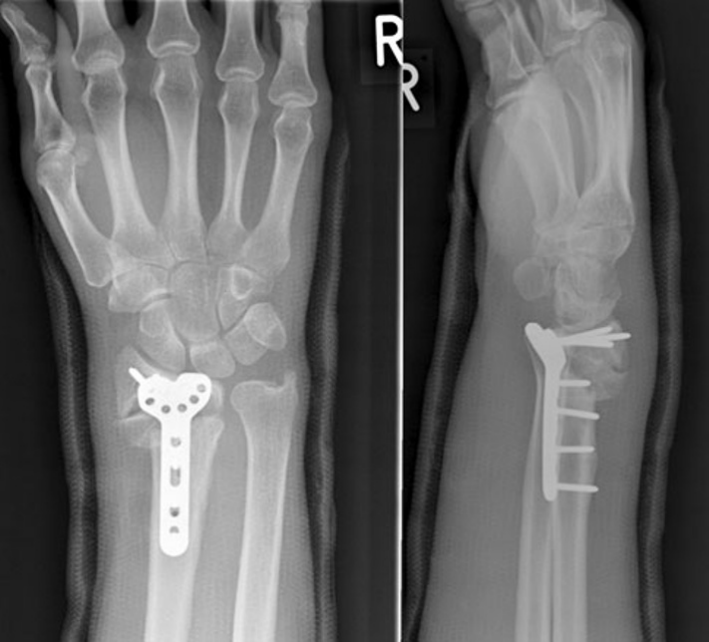
Radiographs at 6 weeks after the second revision procedure (with bone graft)

**Fig. 6:**
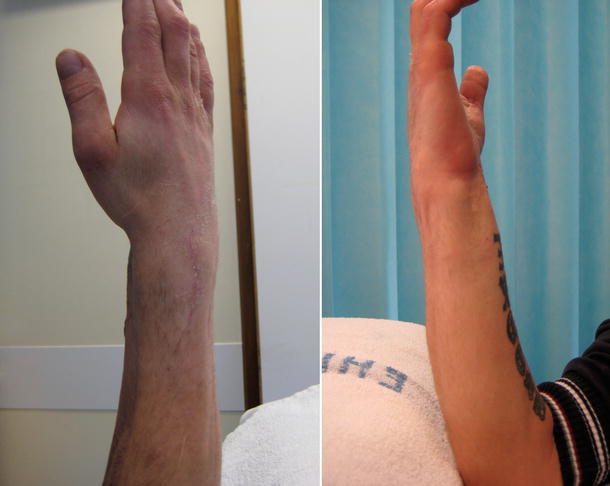
Clinical examination at 6 weeks after the second revision

**Fig. 7:**
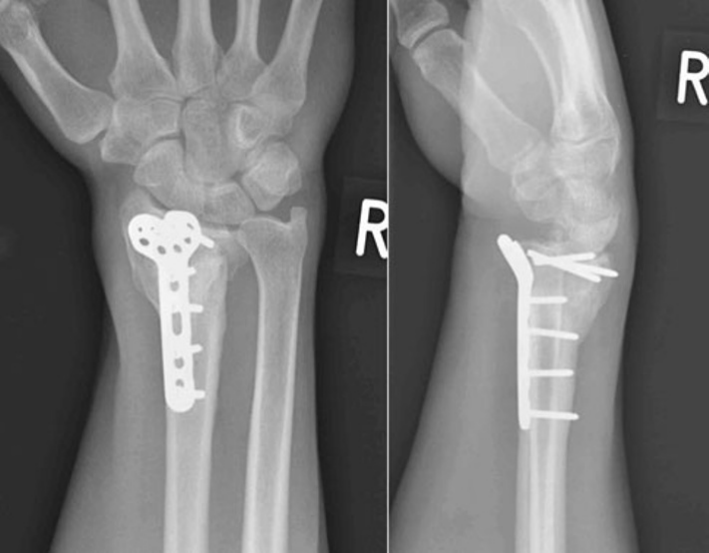
Radiographs at 1 year after second revision

A second revision fixation was performed with another 2.4 mm LCP Distal Radius Plate (Synthes^™^, Solothurn, Switzerland). The patient consented to iliac crest bone graft this time. The wrist was re-immobilised in a plaster cast post-operatively. He was non-tender over the distal radius when examined at 6 weeks after this second revision procedure, and all the wounds had healed well (Figs. 5, 6). Immobilisation was continued for another 4 weeks. At the last follow-up at 12 months after the second revision fixation, the fracture had united clinically and radiologically. The three distal screws had, however, broken, without the plate coming off the bone (Fig. 7). His flexion–extension (FE) arc was 90°, with radial-ulnar (RU) deviation arc of 30°; all movements were pain-free. His grip strength was 70% of the opposite wrist. He had regained employment and had cut down on smoking. It was decided to leave the metal-ware in situ, as it was asymptomatic, and the patient was discharged from follow-up.

## Discussion

Locking plates are being increasingly used for the stabilisation of distal radius fractures [1]. The advantages include a stable osteosynthesis, especially in osteoporotic or multifragmented bone, with earlier joint mobilisation and return to function. The design of these locking plates is increasingly being refined, making them pre-bent, near-anatomical and low-profile to conform to the palmar aspect of the wrist and forearm. Various complications of this mode of fracture fixation have been described to-date [2–4] including extensor and flexor tendon ruptures, loss of reduction and non-union, carpal tunnel syndrome, and vascular complications. Plate breakage is a comparatively rarer complication of palmar locking plate fixation [5]. A literature search has revealed three case reports of plate breakage [6–8] and one case of breakage of the distal screws [9]. Both biological and biomechanical factors have been implicated.

De Baere et al. [6] noted breakage of a 3.5 mm T-type LCP (Mathys^™^ Medical Ltd., Bettlach, Switzerland) nearly 14 weeks after the fixation of a distal radius fracture. They cited diabetes as a biological cause contributing to this, as it impairs capillary vascularity and hence fracture healing. Inadequate reduction in the palmar cortex and suboptimal contact between it and the plate contributed to increased load transmission through the implant and eventually caused plate breakage. Imade et al. [7] have reported on a Matrix^®^ LCP (Stryker^™^, Kalamzoo, MI, USA), which broke only 1 week after surgery. They theorised that placing the most distal screw in the proximal fragment too near to the fracture site accentuated the mechanical stress in that area, leading to plate failure. Yukata et al. [8] reported breakage of a Matrix^®^ Smartlock Titanium plate (Stryker Leibinger^™^, Freiburg, Germany), which had been implanted to stabilise an osteoporotic and multifragmented fracture. They postulated that increased stress from early weight bearing was responsible for failure.

Plate breakages seem to occur in the vicinity of unfilled screw holes, adjacent to the fracture site [6–8]. A locking mechanism on the screws implies that they cannot loosen out of the plate, increasing the load on the plate itself. If the biological environment is not conducive to fracture healing, the forces through the implant are exaggerated. Additionally, this high-stress area corresponds to the ‘bend’ on these pre-contoured plates. Osada et al. [9] have demonstrated that plates fail in palmar apex angulation. ‘Stress concentration’ due to the transmission of excessive loads through unsupported plate in this small area could thus possibly explain this mode of plate breakage. It is corroborated by Trease et al’s experiment [10], in which they axially loaded both locked and non-locked plate constructs in osteotomy models, and found that these always failed through the unfilled screw holes at the osteotomy site.

**Fig. 8:**
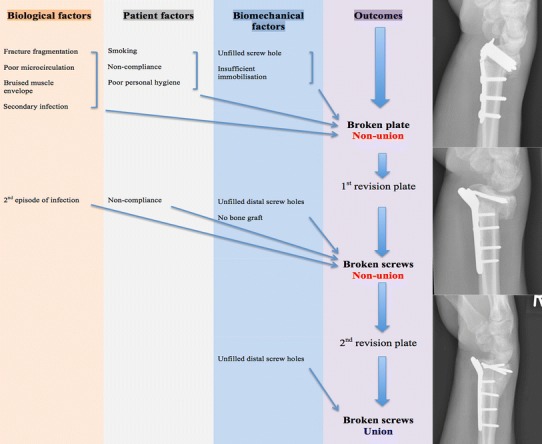
Suggested causes for sequential failures of the three plates

The failure of the original procedure in the presented case seems multifactorial (Fig. 8). These possible causes include patient factors (non-compliance, smoking and poor personal hygiene), biological factors (fracture fragmentation, impaired circulation, bruised muscular envelope and secondary infection) and mechanical factors (no bone graft, unfilled screw holes and insufficient immobilisation). The original plate was applied across a multifragmented fracture, surrounded by bruised muscular mass. The skin graft and its subsequent infection precluded immobilisation in a full cast. The patient did not adhere to non-weight-bearing instructions. Mechanical stresses were therefore able to build up in the vicinity of the unfilled Combi-hole, surrounded by a biologically unfavourable environment. This presumably led to the first plate failure and non-union.

Once the infection became ‘deep-seated’ and refractory to antimicrobial therapy, the fracture biology was impaired significantly. Bony integrity was compromised because of the multifragmentation, and the patient had refused to have a bone graft. He was also non-compliant again with weight-bearing instructions. Thus, the first revision plate was subject to exaggerated stresses again. Since the proximal portion of the locking plate was now more stable with all holes filled with screws, the stresses now concentrated at the four distal screws, causing them to break. If a bone graft had been used at that stage, and all the available screw holes for the distal fragment had been filled up, the load transmission would intuitively have been more evenly distributed, thereby lessening the stress concentration. Unfilled screw holes in the distal fragment have previously been suggested as the mechanical factor responsible for screw breakage in a Aculock^®^ locking plate (Acumed^™^, Hillsboro, OR, USA) [11]. This also explains the breakage of the screws in the second revision plate. However, improved biological and patient factors allowed the fracture to unite.

We feel that patient selection is important to ensure success of osteosynthesis. Additionally, both biological and biomechanical factors need to be considered when deciding on the type and mode of plate, utilisation of use of bone graft and the duration of immobilisation.
